# Ca^2^^+^-dependent rapid uncoupling of astrocytes upon brief metabolic stress

**DOI:** 10.3389/fncel.2023.1151608

**Published:** 2023-10-11

**Authors:** Sara Eitelmann, Katharina Everaerts, Laura Petersilie, Christine R. Rose, Jonathan Stephan

**Affiliations:** Institute of Neurobiology, Heinrich Heine University Düsseldorf, Düsseldorf, Germany

**Keywords:** gap junctions, ischemia, isopotentiality, calcium, pH, sodium, neocortex

## Abstract

Astrocytic gap junctional coupling is a major element in neuron–glia interaction. There is strong evidence that impaired coupling is involved in neurological disorders. Reduced coupling was, e.g., demonstrated for core regions of ischemic stroke that suffer from massive cell death. In the surrounding penumbra, cells may recover, but recovery is hampered by spreading depolarizations, which impose additional metabolic stress onto the tissue. Spreading depolarizations are characterized by transient breakdown of cellular ion homeostasis, including pH and Ca^2+^, which might directly affect gap junctional coupling. Here, we exposed acute mouse neocortical tissue slices to brief metabolic stress and examined its effects on the coupling strength between astrocytes. Changes in gap junctional coupling were assessed by recordings of the syncytial isopotentiality. Moreover, quantitative ion imaging was performed in astrocytes to analyze the mechanisms triggering the observed changes. Our experiments show that a 2-minute perfusion of tissue slices with blockers of glycolysis and oxidative phosphorylation causes a rapid uncoupling in half of the recorded cells. They further indicate that uncoupling is not mediated by the accompanying (moderate) intracellular acidification. Dampening large astrocytic Ca^2+^ loads by removal of extracellular Ca^2+^ or blocking Ca^2+^ influx pathways as well as a pharmacological inhibition of calmodulin, however, prevent the uncoupling. Taken together, we conclude that astrocytes exposed to brief episodes of metabolic stress can undergo a rapid, Ca^2+^/calmodulin-dependent uncoupling. Such uncoupling may help to confine and reduce cellular damage in the ischemic penumbra *in vivo*.

## 1. Introduction

Astrocytes in the mouse brain are coupled via gap junctions mainly composed of connexin (Cx) 43 and Cx30, forming extensive functional syncytia (Giaume et al., 2021; Stephan et al., 2021). This coupling allows the intercellular diffusion of small molecules and ions (e.g., K^+^, Na^+^, Ca^2+^) and metabolites (e.g., glucose, lactate). Gap junctions also play an important role in the delivery of energy metabolites to neurons (Rouach et al., 2008). Furthermore, they facilitate the uptake of excess extracellular glutamate and K^+^ released upon synaptic activity, thereby regulating neuronal excitability and synaptic plasticity (Pannasch et al., 2011; Hosli et al., 2022). Notably, astrocyte coupling is not static, but is dynamically adjusted to the activity and energy demand of neighboring neurons (Rouach et al., 2008). Astrocytic gap junctional coupling thus emerges as a major element in neuron–glia interaction and communication in the healthy brain (Giaume et al., 2021; Mazaud et al., 2021).

Many pathological conditions, however, result in an uncoupling of astrocytes. For example, coupling is significantly reduced in epilepsy, resulting in an impaired clearance and buffering of K^+^. This suggests a causative involvement of an impaired gap junctional coupling in the development of this disease (Boison and Steinhauser, 2018). Uncoupling of gap junctions was also demonstrated in astrocytes exposed to conditions of prolonged metabolic failure, which mimic acute conditions in the core region of an ischemic stroke (Vera et al., 1996; Cotrina et al., 1998; Xu et al., 2010; Lee et al., 2016). The ischemic core is characterized by a dramatic drop in blood flow and an energy supply that is too low to maintain the activity of ion pumps and secondary-active transporters (Struyk, 2005). This results in a breakdown of ion gradients, followed by irreversible cell damage (Rossi et al., 2007; Moskowitz et al., 2010; van Putten et al., 2021).

In the adjacent penumbra, blood flow is reduced but high enough to initially maintain the viability of neurons and astrocytes. Full recovery is possible, provided that reperfusion is regained in time (Moskowitz et al., 2010). Recovery in the penumbra is, however, jeopardized by waves of spreading depolarizations. These invade the penumbra from the ischemic core, aggravating hypoxia and thereby playing an important role in the gradual expansion of the ischemic core (Dirnagl et al., 1999; Rossi et al., 2007; Lauritzen et al., 2011; Hinzman et al., 2014; Dreier and Reiffurth, 2015). Hallmarks of spreading depolarizations are reversible accumulations of extracellular glutamate and K^+^. In both, neurons and astrocytes, this is accompanied by an increase in intracellular Ca^2+^ and Na^+^ concentration ([Ca^2+^]_i_, [Na^+^]_i_), by an acidification, as well as a decrease in cellular ATP (Pietrobon and Moskowitz, 2014; Dreier and Reiffurth, 2015; Rakers and Petzold, 2017; Gerkau et al., 2018). Notably, gap junctional conductance is directly sensitive to changes in intracellular pH (pH_i_) and [Ca^2+^]_i_ (Giaume et al., 2021). However, it is still unclear, if transient ionic alterations such as those that are characteristic for the ischemic penumbra and subject to spreading depolarizations will result in an immediate change in astrocyte coupling.

To address this question, we examined the effects of brief metabolic stress on gap junctional coupling between astrocytes in acute tissue slices of the mouse neocortex in an *in situ* model mimicking features of the ischemic penumbra, e.g., decrease of ATP levels, [Na^+^]_i_ increase, and intracellular acidification (Pietrobon and Moskowitz, 2014; Gerkau et al., 2018; Lerchundi et al., 2019; Eitelmann et al., 2022). Gap junctional coupling was assessed by recording the syncytial isopotentiality, a direct measure of coupling strength (Stephan et al., 2021). In addition, coupling was analyzed by tracer loading and by imaging of the intercellular spread of induced Na^+^ elevations. Imaging of astrocytic pH_i_ and [Ca^2+^]_i_ was performed to address the mechanisms triggering the observed changes in gap junctional coupling. Our results demonstrate that neocortical astrocytes *in situ* uncouple upon strong Ca^2+^ loads and subsequent activation of calmodulin evoked by metabolic stress.

## 2. Materials and methods

### 2.1. Ethical approval

The present study was carried out in strict accordance with the institutional guidelines of the Heinrich Heine University Düsseldorf, as well as the European Community Council Directive (2010/63/EU). All procedures were reported to, and approved by, the Animal Welfare Office of the Animal Care and Use Facility of the Heinrich Heine University Düsseldorf (institutional act number: O52/05). Based on the recommendations of the European Commission (Close et al., 1997), mice were anesthetized with CO_2_, rapidly decapitated, and their brains were quickly removed. According to the German Welfare Act (TSchG; section 4, paragraph 3), no additional approval was required for *post mortem* removal of brain tissue.

### 2.2. Preparation of acute tissue slices and salines

Experiments were carried out using acute parasagittal tissue slices containing layer II/III of the somatosensory cortex from Balb/C mice of both sexes at postnatal days 14–21. Immediately after dissection, brains were transferred into ice-cold (3 ± 1°C) preparation saline containing (in mM): 130 NaCl, 2.5 KCl, 1.25 NaH_2_PO_4_, 26 NaHCO_3_, 0.5 CaCl_2_, 6 MgCl_2_, and 10 glucose; pH 7.4, 310 ± 5 mOsm; bubbled with carbogen (95% O_2_, 5% CO_2_). Thereafter, 250 μm-thick neocortical slices were cut using a vibratome (HM650V, Microtome, Thermo Fisher Scientific, Waltham, MA, USA). For *a priori* identification of astrocytes, slices were incubated for 20 min at 34°C in preparation saline containing 0.5–1 μM sulforhodamine (SR) 101 (Kafitz et al., 2008). Thereafter, slices were incubated for 10 min at 34°C in SR101-free standard artificial cerebrospinal fluid (ACSF) containing (in mM): 130 NaCl, 2.5 KCl, 1.25 NaH_2_PO_4_, 26 NaHCO_3_, 2 CaCl_2_, 1 MgCl_2_, and 10 glucose; pH 7.4, bubbled with carbogen. Afterwards, slices were stored in ACSF at room temperature (21 ± 1°C) until they were used for experiments, which were also performed at room temperature. During experiments, slices were constantly perfused with ACSF at a rate of 2.5 ml/min.

All chemicals were purchased from Merck/Sigma-Aldrich (St. Louis, MO, USA) or AppliChem (Darmstadt, Germany), if not stated otherwise. We utilized an *in situ* model that mimics features of the ischemic penumbra undergoing a spreading depolarization, e.g., decrease of ATP levels, [Na^+^]_i_ increase, and intracellular acidification (Pietrobon and Moskowitz, 2014; Gerkau et al., 2018; Lerchundi et al., 2019; Eitelmann et al., 2022). To this end, tissue slices were perfused for 2 min with glucose-free ACSF containing the cytochrome C inhibitor sodium azide (NaN_3_; 5 mM) and the non-metabolizable glucose analog 2-deoxyglucose (2-DG; 2 mM) to inhibit cellular ATP production (“chemical ischemia”; Gerkau et al., 2018; Lerchundi et al., 2019; Eitelmann et al., 2022). Gap junctional communication was blocked by perfusing slices with carbenoxolone (CBX; 100 μM). The influence of pH_i_ on gap junctional coupling was investigated by perfusing slices for either 2 or 5 min with an ACSF containing a lower HCO_3_^−^ concentration ([HCO_3_^−^]_e_; 3.6 mM, pH 6.4; *cf.* Wallraff et al., 2006). To reduce Ca^2+^ entry into astrocytes, KB-R7943 (NCX inhibitor, 50 μM, Abcam, Cambridge, UK), DL-AP5 (NMDA receptor antagonist, 100 μM; StressMarq Biosciences, Cadboro Bay Village, Canada), and HC-067047 (TRPV4 antagonist, 10 μM) were added to the saline. Calmodulin - a known gap junction modulator (Zou et al., 2014; Peracchia, 2020) - was inhibited using trifluoperazine (TFP, 10 μM, Biomol/Cayman, Ann Arbor, MI; USA). Bath application of blockers was started 15 min prior to and maintained throughout the entire duration of experiments.

### 2.3. Electrophysiology

Patch-clamp experiments were performed at an upright microscope (E600FN or FN1, Nikon, Tokyo, Japan), equipped with (infrared) differential interference contrast optics including a 60x water immersion objective (Fluor 60x/1.00 W, DIC H/N2, ∞/0 WD 2.0, Nikon) and an (infrared) video camera (XC-ST70CE, Hamamatsu Photonics, Herrsching, Germany or TruChrome Metrics, Tucsen Photonics Co., Gaishan Town, Cangshan Area, Fuzhou, Fujian, China). Electrophysiological recordings were performed using an EPC10 amplifier and “PatchMaster” software (Harvard Bioscience/HEKA Elektronik, Lambrecht, Germany). Patch pipettes were pulled from borosilicate glass capillaries (GB150(F)-8P, Science Products, Hofheim am Taunus, Germany) at a vertical puller (PC-10 Puller, Narishige International, London, UK) and had a resistance of 3.5–5.0 MΩ. The standard pipette solution contained (in mM): 116 K-methanesulfonate, 32 KCl, 10 HEPES (N-(2-hydroxyethyl)piperazine-*N′*-2-ethanesulfonic acid), 10 NaCl, 4 Mg-ATP, and 0.4 Na_2_-GTP; pH 7.3. The offset potential was corrected. Data were analyzed using “OriginPro 2021” (OriginLab Corporation, Northampton, MA, USA).

Syncytial isopotentiality was determined as previously described by the Zhou lab (Ma et al., 2016; Kiyoshi et al., 2018). To this end, a K^+^-free pipette solution (0 mM K^+^) was used in which K-methanesulfonate and KCl were substituted with an equimolar amount of Na-methanesulfonate and NaCl, respectively. After achieving a GΩ seal, the recording was switched to current-clamp mode and the membrane patch was ruptured by applying negative pressure. This resulted in a rapid hyperpolarization in the recorded potential reflecting the current membrane potential (*E_M_*) of the cell. Subsequent dialysis of the cell with 0 mM K^+^ led to a decline in *E_M_* to a new steady state level. The latter represents a direct measure of gap junctional coupling, i.e., the ability to equalize the *E_M_* of astrocytes within the functional syncytium (“isopotentiality”). Whole-cell recordings were discarded when input resistance exceeded 30 MΩ.

Changes in astrocytic *E_M_* in response to brief metabolic stress were determined by cell-attached recordings as described before (Eitelmann et al., 2022). Pipettes were filled with standard ACSF and the remaining offset potential was corrected. In cell-attached recordings, the input resistance should be at least 100-fold higher than the membrane resistance (*R_M_*) to ensure reliable measurement of *E_M_* (Perkins, 2006). As *R_M_* of mature astrocytes is about 10 MΩ (Kafitz et al., 2008), an input resistance higher than 1 GΩ was required for cell-attached recordings.

### 2.4. Tracer coupling

Visualization of astrocytic gap junctional coupling by tracer loading was done as described earlier (Eitelmann et al., 2019, 2020). Briefly, an astrocyte was recorded in whole-cell configuration for 20 min using a pipette solution that contained the gap junction-impermeable dye Alexa Fluor (AF) 568 (100 μM, Thermo Fisher Scientific/Invitrogen, Carlsbad, CA, USA) and the gap junction-permeable tracer neurobiotin (2%, Vector Laboratories, Burlingame, CA, USA) to visualize the patched astrocyte as well as its coupled neighbors, respectively. Afterwards, slices were fixed at 4°C with 4% paraformaldehyde overnight. All consecutive steps were carried out at room temperature. Slices were washed three times for 10 min in phosphate buffered saline (PBS) containing NaCl, Na_2_HPO_4_*2 H_2_O, and NaH_2_PO_4_*H_2_O (pH 7.4). Membrane permeabilization was achieved by incubation in 0.25% triton X-100 for 30 min, followed by a wash with PBS. For detection of neurobiotin, slices were then incubated with avidin AF488 (50 μg/ml, Invitrogen) for 3 h. Finally, slices were washed in PBS, transferred on microscope slides (VWR, Radnor, PA, USA) and embedded in mounting medium Mowiol.

Staining were documented using a confocal system (Eclipse C1, Nikon) equipped with an upright microscope (E600FN, Nikon) and a 60x oil immersion objective (Plan Apo VC 60x/1.40 Oil, ∞/0.17 WD 0.13, Nikon). Confocal images were acquired using “EZ-C1 FreeViewer” software (Nikon). Fluorophores were excited and detected as follows (excitation wavelength/filtered emission wavelength): AF488 (488 nm/515/30 nm) and AF568 (543 nm/605/75 nm). Images were further processed using “FIJI” software (Schindelin et al., 2012).

### 2.5. Imaging of [Na^+^]_i_, pH_i_, or [Ca^2+^]_i_

To measure changes in [Na^+^]_i_, pH_i_, or [Ca^2+^]_i_, tissue slices were loaded with the membrane-permeable forms of SBFI (SBFI-AM; sodium-binding benzofuran isophthalate-acetoxymethyl ester, 116.7 μM; Teflabs, Austin, USA), BCECF (BCECF-AM; 2′,7’-Bis-(2-Carboxyethyl)-5-(and-6)-Carboxyfluorescein-acetoxy-methyl ester, 125 μM; A.G. Scientific, San Diego, CA, USA) or OGB-1 (OGB-1-AM, Oregon Green 488 BAPTA-1-acetoxymethyl ester; 111 μM; Invitrogen), respectively. Wide field imaging was performed using an upright microscope (Eclipse FN1, Nikon) equipped with a 40x water immersion objective (Fluor 40×/0.8 W, DIC M/N2, ∞/0 WD 2.0, Nikon) and fluorophores were excited using a Polychrome V monochromator (Thermo Fisher Scientific/FEI, Planegg, Germany). Astrocytes were identified by additional SR101-labeling (excitation at 575 nm, emission collected >590 nm). SBFI was excited at 400 nm and its fluorescence was detected above ~430 nm (409 beam splitter and 510/84 emission filter; Gerkau et al., 2018). BCECF was excited at 458 (isosbestic wavelength) and 488 nm (pH-sensitive wavelength), and its emission was recorded between 511 and 563 nm. OGB-1 was excited at 488 nm, and its emission was collected above 505 nm.

Images of SBFI, BCECF, and OGB-1 fluorescence were acquired at 0.5–1 Hz with an ORCA FLASH 4.0LT camera (Hamamatsu Photonics). Fluorescence was collected from regions of interest (ROIs) representing cell bodies of SR101-positive astrocytes. Emission from individual ROIs was background corrected and SBFI and OGB-1 signals were additionally corrected for bleaching by employing “OriginPro 2019/2021.” For BCECF, the fluorescence ratio (F_458_/F_488_) was calculated after background correction. Normalization of fluorescence was performed in MS Excel 2016. *In situ* calibration of SBFI (Michaelis–Menten fit), BCECF (linear fit), and OGB-1 (Michaelis–Menten fit) fluorescence was done as described before (Gerkau et al., 2018; Ziemens et al., 2019; Eitelmann et al., 2022). Changes in [Ca^2+^]_i_ were analyzed immediately at onset of reperfusion, i.e., 2 min after start of chemical ischemia wash-in.

### 2.6. Statistics

Each set of experiments was performed on at least three tissue slices taken from at least three different animals. Before further processing, data were tested for outliers using “WinSTAT” (R. Fitch Software, Bad Krozingen, Germany).

An earlier study provided evidence for the existence of two astrocyte populations in mouse neocortex, responding differently to energy deprivation (Benesova et al., 2009). Data were thus tested for possible classification into *k* clusters using “Matlab” software (Matlab R2022a, The MathWorks, Inc., Portola Valley, CA, USA). Hereby, up to four clusters were allowed, i.e., twice the number of previously reported populations (Benesova et al., 2009). Then, a two-dimensional *k*-means algorithm using the silhouette criterion and a minimal cluster distance of 1 was utilized to determine the optimal number of clusters *k*. Subsequently, data were clustered using the agglomerative hierarchical clustering algorithm. First, the similarity between each pair of data points was computed by determining the absolute distance between these values. The data points were then grouped into a hierarchical cluster tree (dendrogram). In this process, data points that were in close proximity to each other were linked according to their calculated distance. Finally, the beforehand calculated optimal number of clusters *k* was used to define the point at which the hierarchical tree is cut into clusters.

Data were further statistically analyzed using “OriginPro 2021”. Normal distribution was assessed using a Shapiro–Wilk test or Kolmogorov–Smirnov test for a sample size (*n*) of <50 or ≥ 50, respectively (Mishra et al., 2019). In case of normal distribution, independent samples were tested by unpaired Student’s *t*-test or ANOVA test (>2 comparisons), whereas paired samples were tested by paired Student’s *t*-test or repeated measures ANOVA test (>2 comparisons). In the absence of normal distribution, independent samples were tested by Mann–Whitney U test or Kruskal–Wallis *H* test (>2 comparisons), whereas paired samples were tested by Wilcoxon test or Friedman’s ANOVA test (>2 comparisons; Nayak and Hazra, 2011). *p* represents the error probability: *p < 0.05, **p < 0.01, ***p < 0.001. Experimental results are provided as mean ± standard deviation (SD). *n* represents the number of cells (or experiments) per slice per animal. Data illustrated in box plots show single data points (grey diamonds), mean (square), median (horizontal line), SD (box), and min/max (whiskers).

## 3. Results

### 3.1. Isopotentiality of neocortical astrocytes

**Figure 1:**
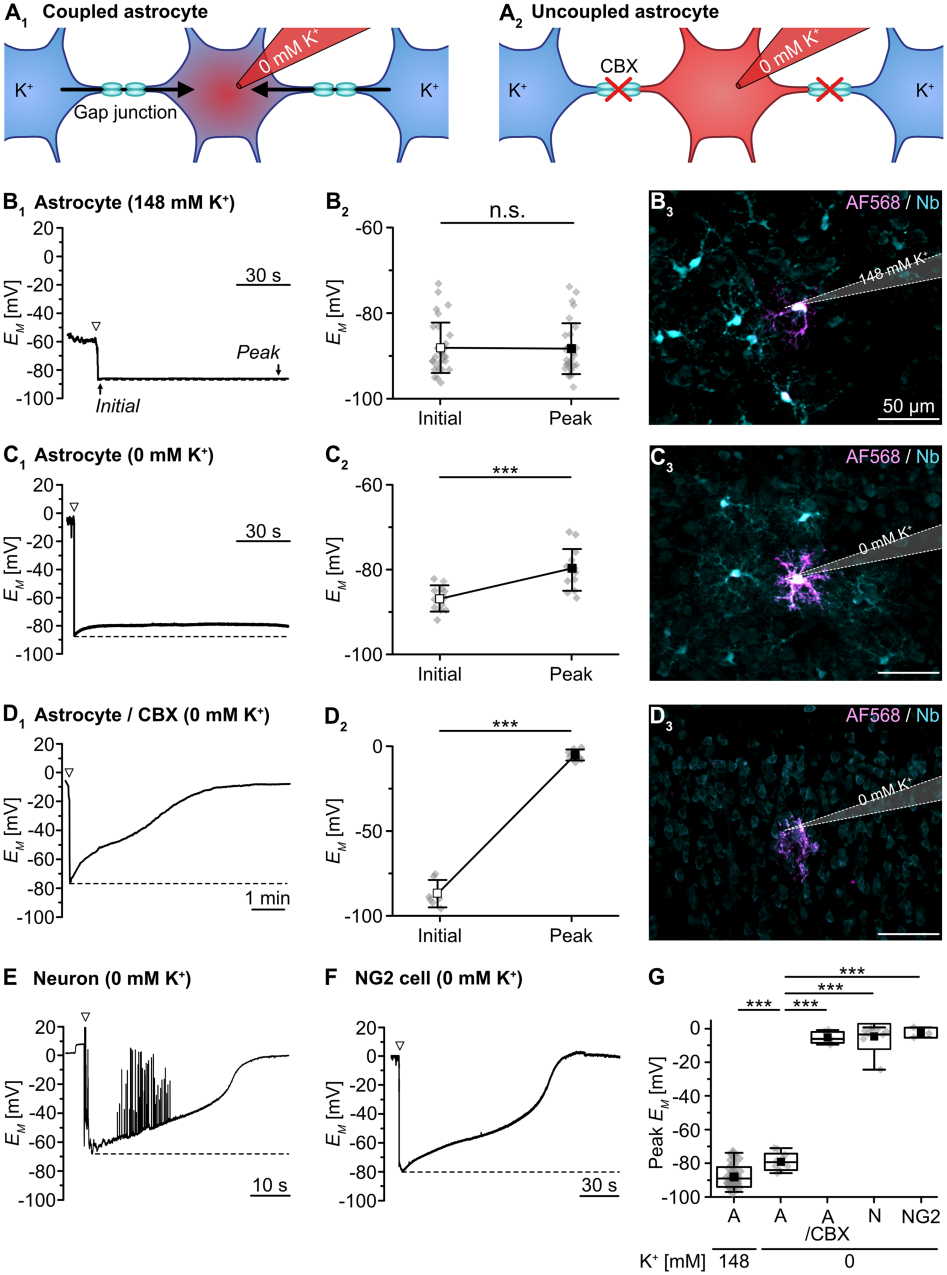
Syncytial isopotentiality of cortical astrocytes. **(A)** Schematic drawing illustrating K^+^ diffusion within the syncytium under different conditions. In whole-cell configuration, the astrocyte is dialyzed by the K^+^-free pipette solution (0 mM K^+^). Under resting conditions, [K^+^]_i_ is expected to be partially buffered within the syncytium **(A**_**1**_**)**. When using the gap junction blocker carbenoxolone (CBX), no buffering occurs and the cytosol is expected to be completely dialyzed **(A**_**2**_**)**. **(B-D)** Experimental evidence for isopotentiality in neocortical astrocytes. *E_M_* was recorded with a pipette solution containing either 148 mM K^+^ **(B)** or 0 mM K^+^ **(C,D)**. With 148 mM K^+^, there was no difference between initial *E_M_* and *E_M_* determined after a few minutes **(B**_**1,2**_**)**. Simultaneous tracer injection of Alexa Fluor 568 (AF568) and neurobiotin (Nb) into the recorded cell revealed coupling to several neighboring cells **(B**_**3**_**)**. Upon patching an astrocyte with 0 mM K^+^, in contrast, initial *E_M_* depolarized to a new steady-state *E_M_* **(C**_**1,2**_**)**. Again, simultaneous tracer injection revealed coupling to several neighboring cells **(C**_**3**_**)**. In a slice pre-incubated with CBX (100 μM), 0 mM K^+^ resulted in a strong depolarization of the patched astrocyte **(D**_**1,2**_**)**. In addition, simultaneous tracer injection labeled only the patched cell **(D**_**3**_**)**. **(E,F)** Patching a pyramidal neuron **(E)** or a presumed NG2 cell **(F)** with 0 mM K^+^ resulted in a strong and multiphasic depolarization. The neuron starts firing action potentials during depolarization **(E)**, whereas the presumed NG2 cell does not generate action potentials **(F)**. The dashed lines in **(B**_**1**_**,C**_**1**_**,D**_**1**_**,E,F)** indicate the initial *E_M_*, directly after rupturing the membrane patch (arrowheads). Dot plots in **(B**_**2**_**,C**_**2**_**,D**_**2**_**)** show individual data points (gray diamonds), mean (squares), and SD (whiskers) of initial *E_M_* and peak *E_M_* determined after a few minutes. **(G)** Box plots illustrating that uncoupled cells exhibited a strongly depolarized peak *E_M_* in isopotentiality recordings as compared to gap junction-coupled astrocytes under control conditions. Shown are individual data points (gray diamonds), mean (squares), median (horizontal line), SD (box), and min/max (whiskers). Significance levels are indicated by asterisks: ***p < 0.001. n.s., not significant.

To measure dynamic changes in gap junctional coupling, we employed an electrophysiological approach based on the isopotentiality of the astrocyte syncytium. Here, diffusion of K^+^, and the ability of the astrocyte syncytium to equalize intercellular differences in *E_M_* via gap junctions, provides a measure of syncytial coupling (Figure 1A). To this end, we first performed whole-cell patch-clamp recordings using a standard pipette solution containing 148 mM K^+^ (Figure 1B). This is close to the estimated intracellular K^+^ concentration ([K^+^]_i_) of astrocytes in layer II/III of the somatosensory cortex (146 mM; *cf.* Eitelmann et al., 2022). Directly upon rupture of the membrane patch, an initial *E_M_* of -87.9 ± 5.8 mV was measured (*n* = 31/20/15). During the ongoing recording, *E_M_* remained stable at -88.1 ± 5.9 mV (*p* = 0.694; Figure 1B_2_). Thus, *E_M_* was essentially unaffected by dialysis of the cytosol with the standard patch pipette solution, which was expected given its near-physiological [K^+^]. Moreover, the experimentally determined *E_M_* was close to the -89.0 mV predicted by the Goldman-Hodgkin-Katz equation (assuming a relative Na^+^/K^+^ permeability of 0.010; *cf.* Eitelmann et al., 2022). Under this resting condition, astrocytes exhibited profound tracer coupling (Figure 1B_3_).

In contrast, using a pipette saline containing 0 mM K^+^ depolarized the astrocytes’ *E_M_* within 10–20 s after breakthrough from an initial *E_M_* of -85.1 ± 3.7 mV to a peak *E_M_* of -78.9 ± 4.4 mV (*n* = 17/15/11, *p* = 9 × 10^−5^; Figures 1C_1,2_). This peak *E_M_* was markedly less positive than expected, as the Goldman-Hodgkin-Katz equation predicts a depolarization to +18.8 mV. The striking difference between the experimentally measured and the theoretically predicted *E_M_* suggests that despite dialysis of 0 mM K^+^ into the patch-clamped cell, its [K^+^]_i_ was largely maintained by equilibration via gap junctional coupling. Accordingly, astrocyte *E_M_* was kept close to physiological levels, though there was small shift in peak *E_M_* (*p* = 1 × 10^−5^; Figure 1G). Like in the preceding experiment, astrocytes were considerably tracer-coupled (Figure 1C_3_).

To confirm that K^+^ exchange within the syncytium limits the depolarizing effect of 0 mM K^+^ in the patch pipette, we repeated the isopotentiality recordings in the presence of the gap junction blocker CBX (100 μM; Figure 1D_1_). When slices were preincubated with CBX, astrocytes depolarized rapidly and *E_M_* essentially collapsed from an initial *E_M_* of -86.7 ± 7.5 mV to a peak *E_M_* of -5.3 ± 3.2 mV (*n* = 7/7/3, *p* = 3 × 10^−7^; Figure 1D_2_). Thus, blocking gap junctions strongly shifts peak *E_M_* (*p* = 1 × 10^−4^; Figure 1G). Tracer coupling from these astrocytes to neighboring cells was not detected, confirming CBX-induced blockage of gap junctions (Figure 1D_3_).

As further proof of concept, we performed isopotentiality recordings with 0 mM K^+^ from cells lacking prominent gap junctional coupling, i.e., from pyramidal neurons in layers II/III and putative NG2 cells. Pyramidal neurons showed a rapid and almost complete depolarization to -4.4 ± 7.8 mV (*n* = 8/8/8, *p* = 1 × 10^−4^; Figure 1E), accompanied by action potential firing after breakthrough and subsequent depolarization, which contrasts astrocyte peak *E_M_* (*p* = 2 × 10^−4^; Figure 1G). SR101-negative cells, exhibiting a small, rounded soma and an initial *E_M_* of -69.0 ± 17.3 mV – properties characteristic for NG2 cells – also depolarized rapidly and strongly to -2.5 ± 3.0 mV after establishing the whole-cell configuration, albeit without generating action potentials (*n* = 3/3/3, *p* = 0.003; Figure 1F), which similarly contrasts astrocyte peak *E_M_* (*p* = 0.008; Figure 1G). As opposed to astrocytes under control conditions, both pyramidal neurons and putative NG2 cells were thus unable to confer isopotentiality.

Taken together, these results confirm that astrocytes in acutely isolated tissue slices of mouse neocortex, in contrast to pyramidal neurons and presumed NG2 cells, confer syncytial isopotentiality. Furthermore, our data show that the ability of individual astrocytes to counteract depolarization induced by dialysis with a K^+^-free pipette solution depends on gap junctional coupling.

### 3.2. Changes in gap junctional coupling upon brief metabolic stress

**Figure 2:**
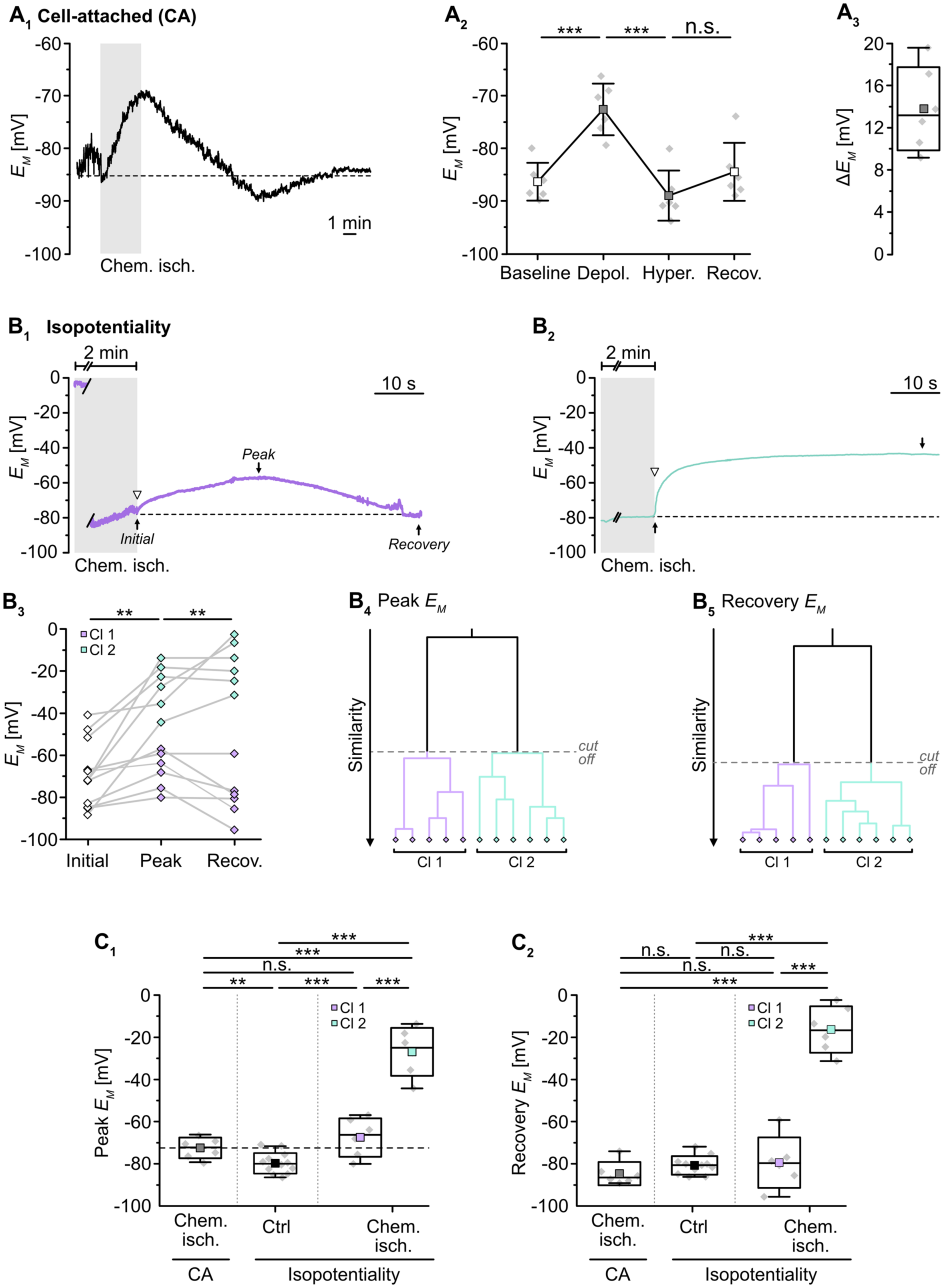
Effect of chemical ischemia on astrocyte syncytial isopotentiality. **(A)** Cell-attached (CA) recording of astrocyte *E_M_*. The recording of a single astrocyte in **(A**_**1**_**)** shows a depolarization followed by a transient hyperpolarization and recovery to the baseline *E_M_* evoked by a 2-minute perfusion with metabolic inhibitors (“chemical ischemia”, gray bar). The dashed line indicates baseline *E_M_*. The plots on the right illustrate changes in *E_M_* **(A**_**2**_**)** and maximal depolarization (Δ*E_M_*; **A**_**3**_). **(B)** Isopotentiality recordings in two different astrocytes exposed to chemical ischemia (gray bars; **B**_**1,2**_). The cell in **(B**_**1**_**)** moderately depolarized from an initial *E_M_* to peak *E_M_* and subsequently recovered, whereas the astrocyte shown in **(B**_**2**_**)** depolarized strongly and remained depolarized. The dashed lines indicate the initial *E_M_* recorded upon membrane rupture (arrowhead). The dot plot illustrates changes in *E_M_* of all cells recorded; paired data are connected by lines **(B**_**3**_**)**. The colored symbols reflect the results obtained from cluster analysis determining the similarity of individual data points, revealing two groups of astrocytes differently responding to 0 mM K^+^ during chemical ischemia **(B**_**4,5**_**)**. Cluster 1 (purple diamonds) showed a moderate depolarization and full recovery, whereas cells from cluster 2 (green diamonds) depolarized to more positive *E_M_* and did not recover **(B**_**3**_**)**. **(C)** Summary of results. **(C**_**1**_**,C**_**2**_**)** Show box plots comparing maximal changes in peak *E_M_* and recovery, respectively, in response to chemical ischemia in cell-attached mode (CA, see **A**_**2**_) and in isopotentiality recordings. “Ctrl”: peak *E_M_* of astrocytes in isopotentiality measurements under control conditions (see Figure 1C_2_). The dashed line in **(C**_**1**_**)** highlights the chemical ischemia-induced mean depolarization recorded in cell-attached mode. Box plots in **(A**_**3**_**,C**_**1**_**,C**_**2**_**)** show individual data points (gray diamonds), mean (squares), median (horizontal line), SD (box), and min/max (whiskers). Significance levels are indicated by asterisks: **p < 0.01, ***p < 0.001. n.s., not significant.

Next, we employed isopotentiality recordings to study changes in gap junctional coupling upon brief metabolic stress. The latter was induced by chemical ischemia, an *in situ* model mimicking features of the ischemic penumbra undergoing spreading depolarizations, e.g., decrease of ATP levels, [Na^+^]_i_ increase, and intracellular acidification (Pietrobon and Moskowitz, 2014; Gerkau et al., 2018; Lerchundi et al., 2019; Eitelmann et al., 2022). Slices were perfused for 2 min with a glucose-free saline that additionally contained 5 mM NaN_3_ and 2 mM 2-DG. Importantly, chemical ischemia *per se* causes a depolarization of astrocytes (e.g., Eitelmann et al., 2022), which will overlap with any changes in *E_M_* induced by isopotentiality recordings with 0 mM K^+^. Therefore, we first performed cell-attached recordings to determine the magnitude of these changes (Figure 2A_1_). Upon chemical ischemia, astrocytes depolarized from a baseline level of -86.4 ± 3.6 mV to -72.6 ± 4.9 mV (*p* = 2 × 10^−7^, *n* = 6/6/4; Figures 2A_2,3_). This peak depolarization was reached 28 ± 22 s after the onset of reperfusion with standard ACSF. Subsequently, astrocytes hyperpolarized to -89.1 ± 4.8 mV (*p* = 9 × 10^−5^) before their *E_M_* recovered to -84.5 ± 5.5 mV (*p* = 1 × 10^−4^), which is close to their initial baseline (Figure 2A_2_).

We next examined changes in the isopotentiality of the astrocyte network upon metabolic stress. Astrocytes were approached with a patch pipette filled with 0 mM K^+^ and a GΩ seal was formed. Subsequently, slices were exposed to chemical ischemia for 2 min and the recording was switched to current-clamp mode to measure *E_M_*. Immediately at onset of reperfusion with standard ACSF, the membrane patch was ruptured, establishing the whole-cell configuration (Figures 2B_1,2_). Subsequently, part of the astrocytes depolarized followed by repolarization, whereas the others showed a more pronounced depolarization and did not recover (Figures 2B_1–3_).

To further characterize this heterogeneous response, cluster analysis was performed. The clustering algorithm analyzed the similarity of individual data points and corroborated the existence of two astrocyte groups, differing in their response to chemical ischemia in the isopotentiality recordings (Figures 2B_4,5_). Cluster 1 (50% of cells) consisted of astrocytes that exhibited an initial *E_M_* of -75.4 ± 8.8 mV immediately after chemical ischemia induction from which cells then depolarized to a peak *E_M_* of -67.2 ± 9.2 mV (*p =* 0.108) within about 14 s (*n* = 6 cells out of 12/12/10; Figures 2B_1,3_,C_1_). As observed before, *E_M_* returned almost completely to baseline (to -79.3 ± 12.0 mV; Figures 2B_1,3_,C_2_). Notably, the peak depolarization of astrocytes affiliated to cluster 1 in isopotentiality recordings did not differ from that observed in cell-attached recordings (*p* = 0.239; Figure 2C_1_), indicating that the isopotentiality of the astrocytic syncytium was maintained. This suggests that astrocytes of cluster 1 did not undergo a change in gap junctional coupling.

In contrast, astrocytes of cluster 2 (the remaining 50%) depolarized from an initial *E_M_* of -61.9 ± 18.2 mV, which was determined immediately after breakthrough, to an average peak *E_M_* of -26.8 ± 11.4 mV (*p =* 0.002). The peak *E_M_* was reached within about 12 s and was maintained throughout the entire recording (at least 5 min, up to 20 min; *n* = 6 cells out of 12/12/10; Figures 2B_2,3_,C_2_). The depolarization induced by chemical ischemia in isopotentiality recordings was significantly higher than that determined in cell-attached recordings (*p* = 4 × 10^−6^; Figure 2C_1_). This indicates that transient metabolic stress resulted in a rapid reduction in the coupling strength in this cluster.

Taken together, our results demonstrate that neocortical astrocytes differently respond to chemical ischemia. Isopotentiality measurements indicate that the coupling strength was maintained in half of the astrocytes recorded from. In contrast, in the other half of the recordings, astrocytes displayed a rapid, sustained reduction in isopotentiality, indicating an immediate and persistent reduction in functional gap junctional coupling in response to brief metabolic stress.

### 3.3. Intercellular sodium diffusion during brief metabolic stress

Our isopotentiality measurements indicated that in acute tissue slices transient chemical ischemia induces an uncoupling in about 50% of the recorded astrocytes. To further probe for such uncoupling, we analyzed the intercellular spread of Na^+^ between astrocytes, which has been shown to diffuse rapidly through gap junctions (Langer et al., 2012). In addition, we determined the length constant *λ* of intercellular Na^+^ diffusion, which is a measure for the effectiveness of Na^+^ redistribution within the network and ranges from 31 to 46 μm depending on the studied brain region (Langer et al., 2012; Augustin et al., 2016; Moshrefi-Ravasdjani et al., 2017; Wadle et al., 2018).

**Figure 3:**
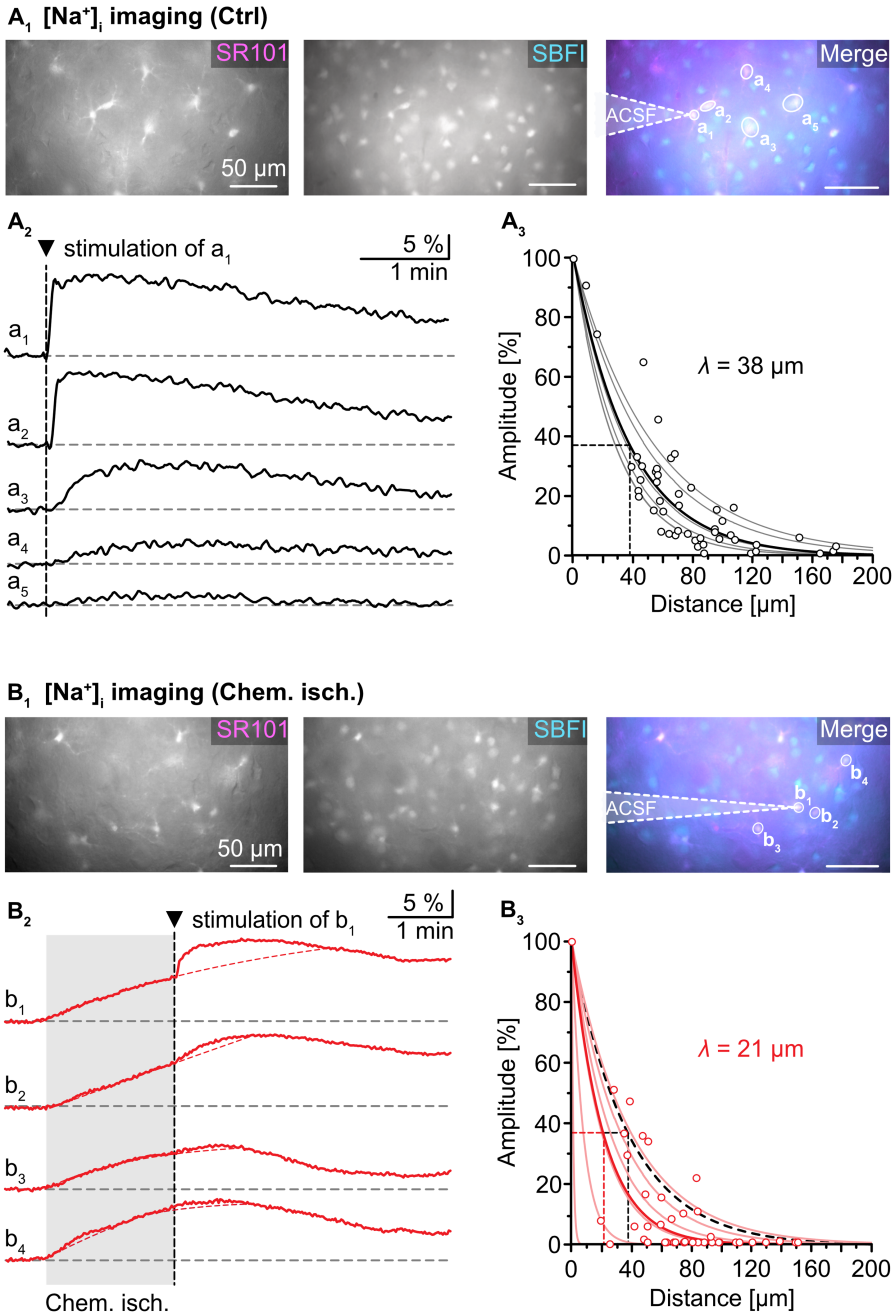
Effect of chemical ischemia on intercellular spread of Na^+^. **(A)** Na^+^ spread during resting conditions. Regions of interest (ROIs) were drawn around somata of SR101-labeled neocortical astrocytes, which were loaded with the Na^+^ indicator SBFI-AM (a_1_–a_5_; **A**_**1**_). Changes in somatic [Na^+^]_i_ of astrocytes a_1_–a_5_ in response to brief electrical stimulation of astrocyte a_1_ (arrowhead, vertical dashed line), depicted as changes in somatic SBFI fluorescence **(A**_**2**_**)**. The graph in **(A**_**3**_**)** shows peak amplitudes of [Na^+^]_i_ increases in individual astrocytes (circles) relative to the amplitude of the directly stimulated cell versus the distance. Data from individual experiments were fit with mono-exponential decay functions (light grey lines). The bold black line shows the decay function of the average length constant (*λ*; *R^2^* = 0.822). **(B)** Na^+^ spread during chemical ischemia. ROIs were drawn around somata of SR101-labeled astrocytes, which were loaded with the Na^+^ indicator SBFI-AM **(B**_**1**_**)**. Two minutes of chemical ischemia (grey box) induced a [Na^+^]_i_ increase in all astrocytes recorded from (b_1_–b_4_; **B**_**2**_). At the time point when switching back to standard ACSF, astrocyte b_1_ was subjected to electroporation (arrowhead, vertical dashed line), inducing an additional [Na^+^]_i_ increase in the stimulated (b_1_) as well as in neighboring cells (b_2_–b_4_). The dashed red lines represent the extrapolated progression of chemical ischemia-induced Na^+^ transients allowing the estimation of electroporation-induced additional Na^+^ load (see text for details). The graph in **(B**_**3**_**)** shows peak amplitudes of [Na^+^]_i_ increases in individual astrocytes (circles) relative to the amplitude of the directly stimulated cell versus the distance. The electroporation was performed right after termination of metabolic stress. The light red lines refer to individual experiments. The bold red line shows the decay function of the average *λ* (*R^2^* = 0.777).

To this end, slices were loaded with the chemical Na^+^ indicator SBFI-AM to record changes in astrocytic [Na^+^]_i_. An individual astrocyte was then approached by an electroporation pipette filled with ACSF and stimulated by a single current pulse (1 ms, 30 mV; Figure 3A_1_). As observed earlier (Langer et al., 2012), this electroporation resulted in an immediate and strong [Na^+^]_i_ increase in the stimulated cell (“a_1_”). Neighboring cells exhibited delayed, smaller increases in [Na^+^]_i_ (“a_2_”–“a_5_”; Figure 3A_2_). The normalized peak amplitude of electroporation-induced elevation of [Na^+^]_i_ decreased mono-exponentially with increasing distance to the simulated astrocyte, with a length constant *λ* ranging from 28 to 54 μm. The average *λ* was 38 ± 11 μm (*R^2^* = 0.822, *n* = 53/6/5, Figure 3A_3_), which is close to values reported from astrocytes in the hippocampus and the auditory brainstem (Langer et al., 2012; Augustin et al., 2016; Wadle et al., 2018). This mono-exponential decay suggests passive spread of Na^+^ from the stimulated cell to its gap junction-coupled neighbors (*cf.* Langer et al., 2012).

Next, astrocytes were exposed to chemical ischemia for 2 min (Figure 3B). As reported before, this results in an astrocytic [Na^+^]_i_ increase (Gerkau et al., 2018; Eitelmann et al., 2022). Immediately upon reperfusion with standard ACSF, a single astrocyte was subjected to electroporation (see cell “b_1_” in Figure 3B_1_). This evoked an additional rapid [Na^+^]_i_ increase in the stimulated astrocyte as well as in neighboring astrocytes (“b_2_”-“b_4_”), which overlapped with the Na^+^ load induced by chemical ischemia (Figure 3B_2_).

To determine the magnitude of the electroporation-induced [Na^+^]_i_ increase independent of the initial chemical ischemia-induced [Na^+^]_i_ increase, the following procedure was employed: First, the second derivative was calculated from each experimental trace. The presence of an inflection point within the first 60 s after electroporation, at which traces bent to the left, was indicative of an additional Na^+^ load. After determination of the inflection point, a mono-exponential fit was created from the original trace that covered the 2-minute rising phase of the chemical ischemia-induced Na^+^ load until the inflection point. This fit was extrapolated to reveal the presumed kinetics of the chemical ischemia-induced Na^+^ load (see red dotted line in Figure 3B_2_). In a final step, the maximum difference between the measured maximum [Na^+^]_i_ and the fit was determined, reflecting the estimated electroporation-induced Na^+^ load.

The peak amplitude of the electroporation-induced Na^+^ load in each cell was normalized to the peak amplitude in the electroporated astrocyte and plotted against the distance from the stimulated astrocyte (Figure 3B_3_). Again, data from individual experiments were fitted with mono-exponential decay curves. Notably, *λ* now ranged from 1 to 40 μm in individual experiments, indicating that in some experiments, a strong reduction in spread of Na^+^ was observed, while in others, Na^+^ spread was essentially unaltered (Figure 3B_3_). Compared to control, the average *λ* was decreased to 21 ± 15 μm (55%, *n* = 51/6/5, *p* = 0.026).

Taken together, these experiments demonstrate that metabolic stress can result in a reduction in the passive spread of Na^+^ between astrocytes. Since gap junctions are the pathway responsible for the intercellular diffusion of Na^+^, this points towards a reduction in gap junctional conductance upon brief metabolic stress.

### 3.4. Influence of pH_i_ on gap junctional coupling

**Figure 4:**
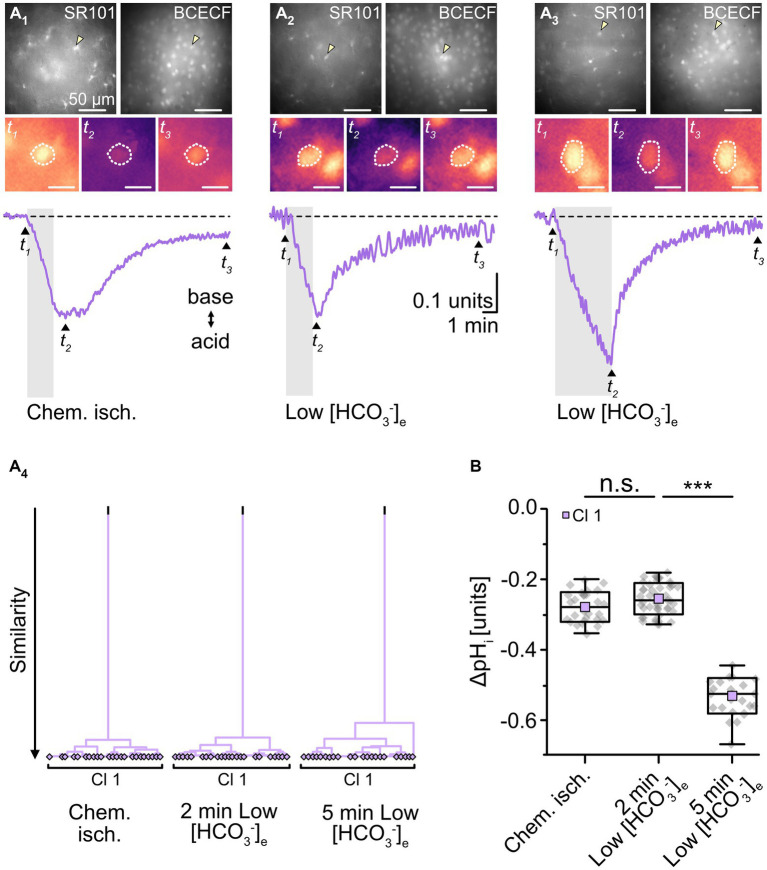
Effect of chemical ischemia on pH_i_. **(A)** Measurement of pH_i_. SR101-labeled astrocytes (**A**_**1–3**_, top left) were loaded with the pH indicator BCECF (**A**_**1–3**_, top right, insensitive wavelength of BCECF). The arrow heads mark exemplary astrocytes, for which somatic BCECF fluorescence is shown before (t_1_), shortly after (t_2_), and longer after (t_3_) respective treatment (**A**_**1–3**_, middle). The analyzed region of interest is encircled by dots. Changes in pH_i_ (depicted as relative changes in BCECF fluorescence) were recorded in response to a 2-minute chemical ischemia (**A**_**1**_, bottom) as well as upon perfusion with low [HCO_3_^−^]_e_ for 2 min (**A**_**2**_, bottom) and for 5 min (**A**_**3**_, bottom). *t*_1–3_ are indicated with labeled arrowheads. The cluster analysis shown in **(A**_**4**_**)** depicts that all pH changes from the different manipulations were grouped into single clusters. **(B)** Summary of results. Box plots illustrate peak acidification in astrocytes induced by the three different manipulations. They show individual data points (gray diamonds), mean (squares), median (horizontal line), SD (box), and min/max (whiskers). Significance levels are indicated by asterisks: ***p < 0.001. n.s., not significant.

We next probed for possible modulators that might mediate the observed changes in gap junctional coupling in neocortical astrocytes. Cerebral ischemia is associated with acidosis in the lesion area (Kraig et al., 1985; Smith et al., 1986; Nedergaard et al., 1991). Additionally, previous work has shown that gap junctional conductance is sensitive to intracellular acidification (Wallraff et al., 2006). Thus, rapid reduction in astrocyte coupling might be mediated by changes in their pH. To this end, we performed wide field imaging experiments employing the pH-sensitive dye BCECF (Figure 4A). Of note and as opposed to the isopotentiality recordings, only one cluster was detected (Figure 4A_4_). Astrocytes acidified by 0.28 ± 0.01 pH units upon chemical ischemia for 2 min (*n* = 29/4/3; Figures 4A_1_,B). Thereafter, pH_i_ recovered to a level slightly more acidic than the initial baseline (Figure 4A_1_).

**Figure 5:**
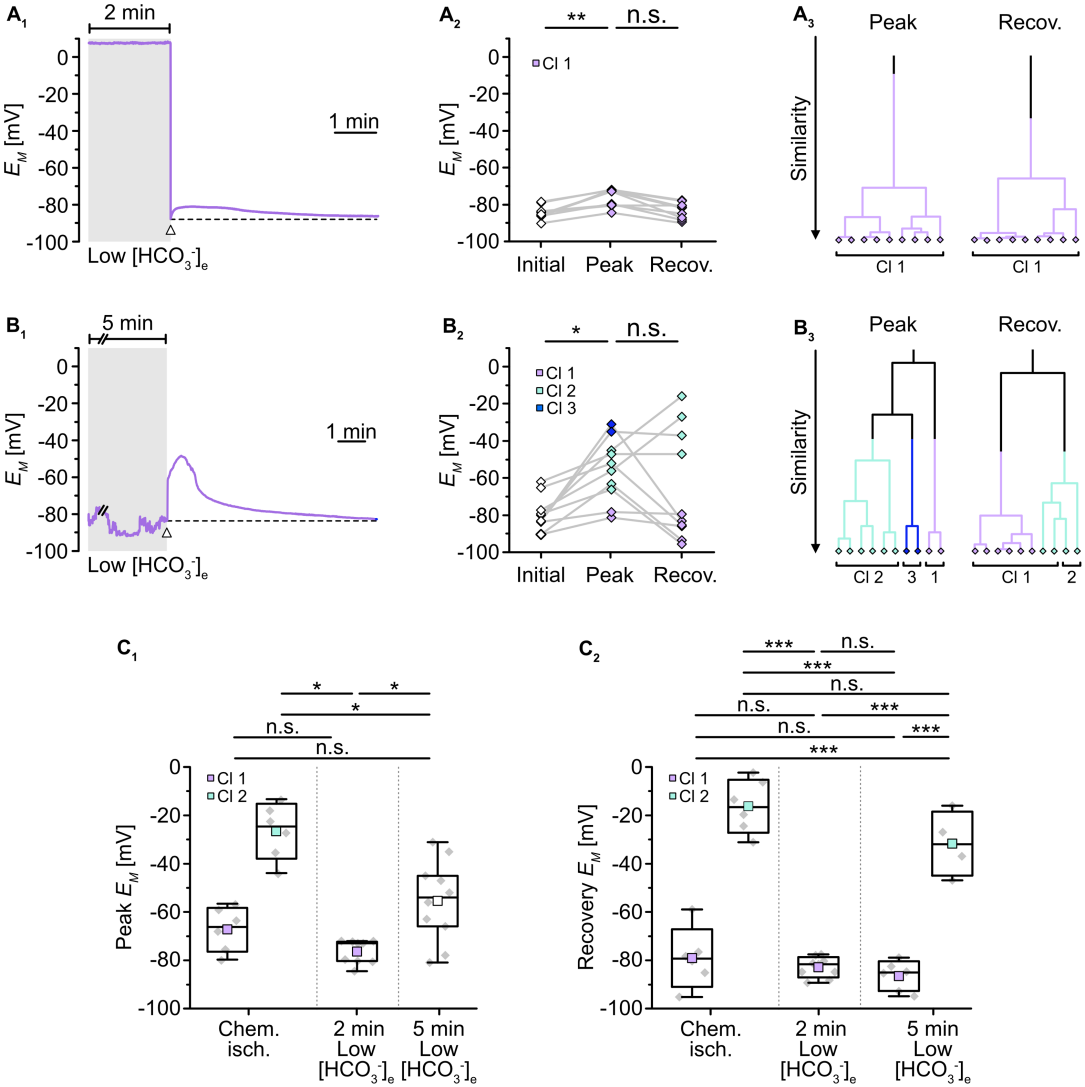
Role of pH_i_ in alteration of gap junctional coupling. **(A,B)** Isopotentiality of astrocytes was recorded in slices treated for 2 min **(A)** or 5 min **(B)** with low [HCO_3_^−^]_e_. Gray bars show the periods when slices were exposed to low [HCO_3_^−^]_e_. The dashed lines in **(A**_**1**_**,B**_**1**_**)** indicate the initial *E_M_* recorded upon membrane rupture (arrowhead). The dot plots in **(A**_**2**_**,B**_**2**_**)** illustrate changes in *E_M_*; paired data points derived from individual cells are connected by lines. The colored symbols reflect the results obtained from cluster analysis determining the similarity of individual data points. The cluster analysis showed that astrocytes responded differently upon 2 min **(A**_**3**_**)** compared to 5 min of low [HCO_3_^−^]_e_ **(B**_**3**_**)**. **(C)** Summary of results. The box plots show peak *E_M_* in isopotentiality recordings **(C**_**1**_**)** and recovery **(C**_**2**_**)** in response to chemical ischemia and application of [HCO_3_^−^]_e_ for 2 and 5 min. They show individual data points (gray diamonds), mean (squares), median (horizontal line), SD (box), and min/max (whiskers). Significance levels are indicated by asterisks: *p < 0.05, **p < 0.01, ***p < 0.001. n.s., not significant.

To mimic such intracellular acidosis, slices were then perfused for 2 min with a saline in which [HCO_3_^−^]_e_ was reduced to 3.6 mM. This indeed resulted in a comparable transient decrease in astrocytic pH_i_ by 0.26 ± 0.01 pH units (*n* = 47/5/3; *p* = 0.367), followed by recovery to baseline in standard ACSF (Figures 4A_2_,B). Astrocytes were affiliated to a single cluster (Figure 4A_4_). Isopotentiality recordings in this low [HCO_3_^−^]_e_ saline showed that the peak *E_M_* of -76.5 ± 4.9 mV (Figures 5A_1,2_), detected about 15–25 s after onset of reperfusion with standard ACSF, was less depolarized compared to chemical ischemia (*n* = 9/8/3, *p* = 0.003; Figure 5C_1_). Again, only one cluster of astrocytes was identified (Figure 5A_3_). Thereafter, all astrocytes fully recovered (Figure 5C_2_). Taken together, these results indicate that a chemical ischemia-like acidification by about 0.26 pH units is not sufficient to mimic the chemical ischemia-induced reduction of gap junctional coupling between astrocytes.

We also extended the perfusion period of low [HCO_3_^−^]_e_ to 5 min, whereupon astrocytes showed a stronger acidification by 0.45 ± 0.05 pH units (*n* = 27/3/2, *p* = 2 × 10^−34^; Figure 4A_3_,B). As before, astrocytes were grouped into only one cluster (Figure 4A_4_). Isopotentiality recordings in which the patch was ruptured after 5 min of low [HCO_3_^−^]_e_ showed a marked depolarization in most of the recorded cells (Figures 5B_1,2_). Here, three clusters could be identified showing small, medium, and large depolarization (Figure 5B_3_). As the clusters that exhibited small and large depolarizations each included only two cells, all data were pooled, yielding an average depolarization to a peak *E_M_* of -55.4 ± 16.8 mV, which was significantly higher than control (*n* = 10/10/4, *p* = 1 × 10^−3^; Figure 5C_1_, *cf*. Figure 2C_1_). The depolarization peaked about 15–30 s after onset of reperfusion with standard ACSF. The peak *E_M_* after 5 min of low [HCO_3_^−^]_e_ was more depolarized compared to 2 min (*p* = 0.009; Figure 5C_1_). Subsequently, six out of ten astrocytes recovered, whereas four astrocytes remained depolarized or depolarized even further (Figures 5B_2_,C_2_). Similar to what was observed upon chemical ischemia, this phenomenon was thus readily reversible in roughly half of the cells, whereas the remainder of cells did not recover.

Taken together, these results demonstrate that an intracellular acidification by about 0.45 pH units causes an immediate and robust reduction in the isopotentiality of astrocytes, indicating a reduction in gap junctional coupling and thereby confirming its well-known dependence on pH_i_. In addition, our results show that a decrease in pH_i_ by about 0.26 pH units does not affect the isopotentiality of astrocytes. This suggests that the intracellular acidification of astrocytes that accompanied brief metabolic stress was not responsible for the observed reduction in gap junctional coupling.

### 3.5. Influence of Ca^2+^ load on gap junctional coupling

**Figure 6:**
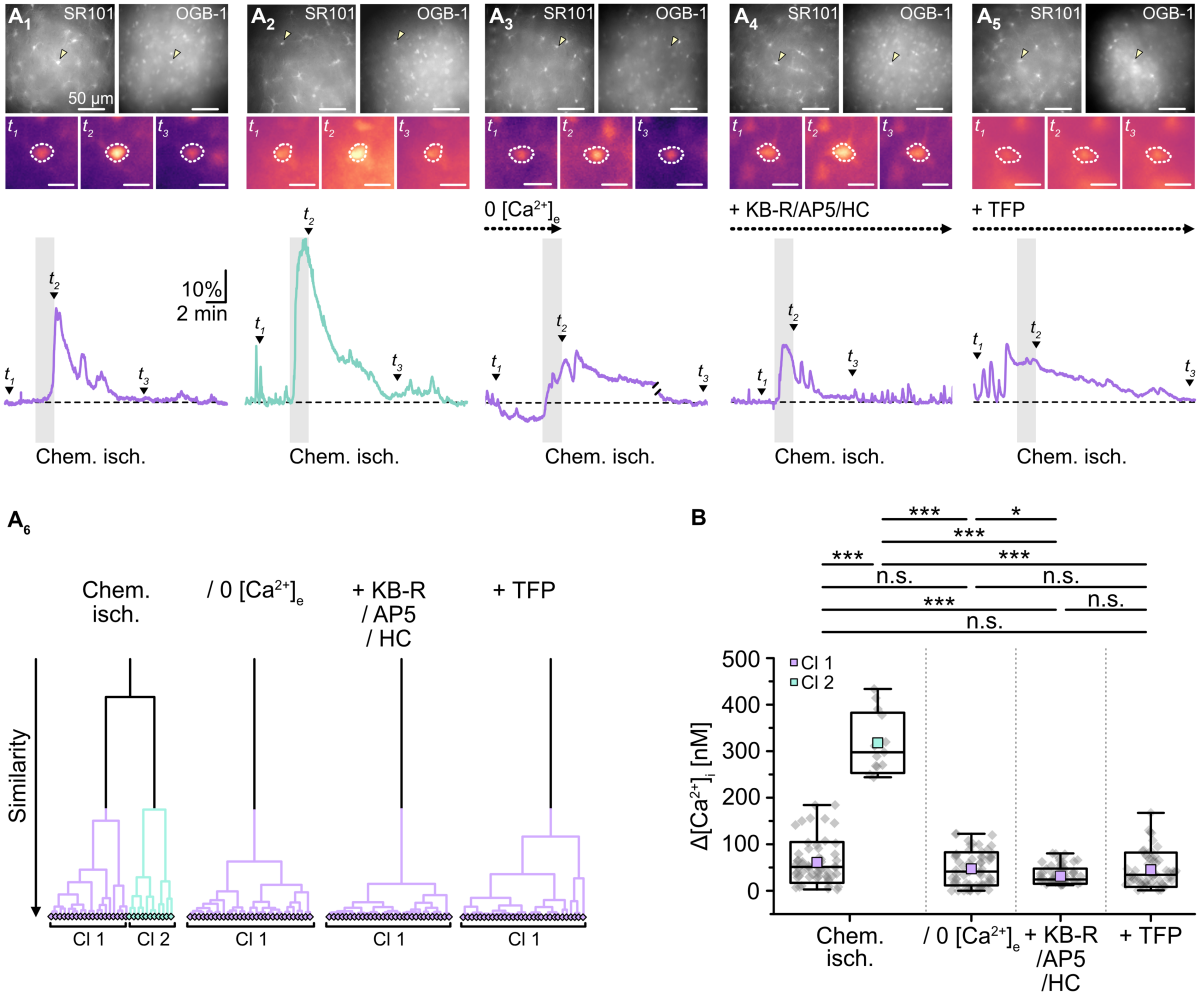
Effect of chemical ischemia on [Ca^2+^]_i_. **(A)** Measurement of [Ca^2+^]_i_. SR101-labeled astrocytes (**A**_**1–5**_, top left) were loaded with the Ca^2+^ indicator OGB-1 (**A**_**1–5**_, top right). The arrow heads mark exemplary astrocytes, for which somatic OGB-1 fluorescence is shown before (*t*_1_), shortly after (*t*_2_), and longer after (*t*_3_) respective treatment (**A**_**1–5**_, middle). The analyzed region of interest is encircled by dots. Changes in [Ca^2+^]_i_ (depicted as relative changes in OGB-1 fluorescence) are shown for individual astrocytes in response to a 2-minute chemical ischemia (**A**_**1,2**_, bottom) as well as a 2-minute chemical ischemia in the nominal absence of extracellular Ca^2+^ (“0 [Ca^2+^]_e_”) (**A**_**3**_, bottom), in the presence of blockers of NCX (KB-R), NMDA receptors (AP5) as well as TRPV4 channels (HC) (**A**_**4**_, bottom), and in the presence of a calmodulin blocker (TFP) (**A**_**5**_, bottom). t_1-3_ are indicated with labeled arrowheads. Whereas cluster analysis revealed the presence of two clusters in control, only one cluster was detected upon reduction of Ca^2+^ influx and inhibition of calmodulin **(A**_**6**_**)**. **(B)** Summary of results. Box plots depict changes in [Ca^2+^]_i_ directly after 2 min of chemical ischemia in control condition, in the absence of [Ca^2+^]_e_, as well as in the presence of blockers of Ca^2+^ influx and calmodulin. They show individual data points (gray diamonds), mean (squares), median (horizontal line), SD (box), and min/max (whiskers). Significance levels are indicated by asterisks: *p < 0.05, ***p < 0.001. n.s., not significant.

In addition to cellular acidification, gap junctions are also modulated by alterations in [Ca^2+^]_i_ and/or an activation of calmodulin, respectively (Lurtz and Louis, 2007; Zou et al., 2014; Peracchia, 2020). To determine whether the observed chemical ischemia-induced reduction of gap junction coupling depends on an induced Ca^2+^ load, we performed imaging experiments with the Ca^2+^-sensitive dye OGB-1 (Figure 6A). As expected from previous work (e.g., Duffy and MacVicar, 1996; Rakers et al., 2017; Gerkau et al., 2018), we found that induction of chemical ischemia caused a rapid, transient increase in astrocytic Ca^2+^ (Figure 6A_1_). Noteworthy, cluster analysis again revealed two groups of astrocytes differing in the strength of their response to chemical ischemia (Figure 6A_6_). Astrocytes of cluster 1 exhibited a moderate [Ca^2+^]_i_ increase of about 60 nM (61 ± 44 nM; range: 3–184 nM; *n* = 61 out of 75/8/6; Figures 6A_1_,B). In astrocytes of cluster 2, the peak amplitude of the [Ca^2+^]_i_ increase was about five times higher, amounting to ~320 nM (318 ± 65 nM; range: 244–434 nM; *n* = 14 out of 75/8/6, *p* = 2 × 10^−9^; Figure 6A_1_,B).

Next, slices were perfused with a saline devoid of Ca^2+^ (0 [Ca^2+^]_e_) to reduce the Ca^2+^ load induced by chemical ischemia. Under these conditions, cluster analysis identified only one group of astrocytes (Figure 6A_6_), which showed an average [Ca^2+^]_i_ increase of about 50 nM (47 ± 36 nM; *n* = 65/6/6; Figures 6A_2_,B). This increase did not differ from that observed in the former cluster 1 (*p* = 0.143) and, consequently, was significantly lower than that of the former cluster 2 (*p* = 2 × 10^−8^, Figure 6B).

A similar result was obtained when perfusing slices with ACSF containing blockers of NCX (KB-R7943, 50 μM), NMDA receptors (DL-AP5, 100 μM) as well as TRPV4 channels (HC-067047, 10 μM) to reduce chemical ischemia-induced Ca^2+^ influx (Rakers et al., 2017; Gerkau et al., 2018). Again, only one cluster was identified (Figure 6A_6_), in which astrocytes showed an [Ca^2+^]_i_ increase by 31 ± 16 nM (*n* = 92/6/5; Figures 6A_2_,B). This was significantly lower than that of both the former cluster 1 and 2 (*p* = 2 × 10^−7^ and *p* = 6 × 10^−9^; Figure 6B). Finally, when we inhibited the gap junction modulator calmodulin by perfusion with TFP (10 μM), chemical ischemia caused a Ca^2+^ load of 46 ± 37 nM (*n* = 56/6/3; Figures 6A_5_,B). Cluster analysis identified only one group of astrocytes (Figure 6A_6_).

**Figure 7:**
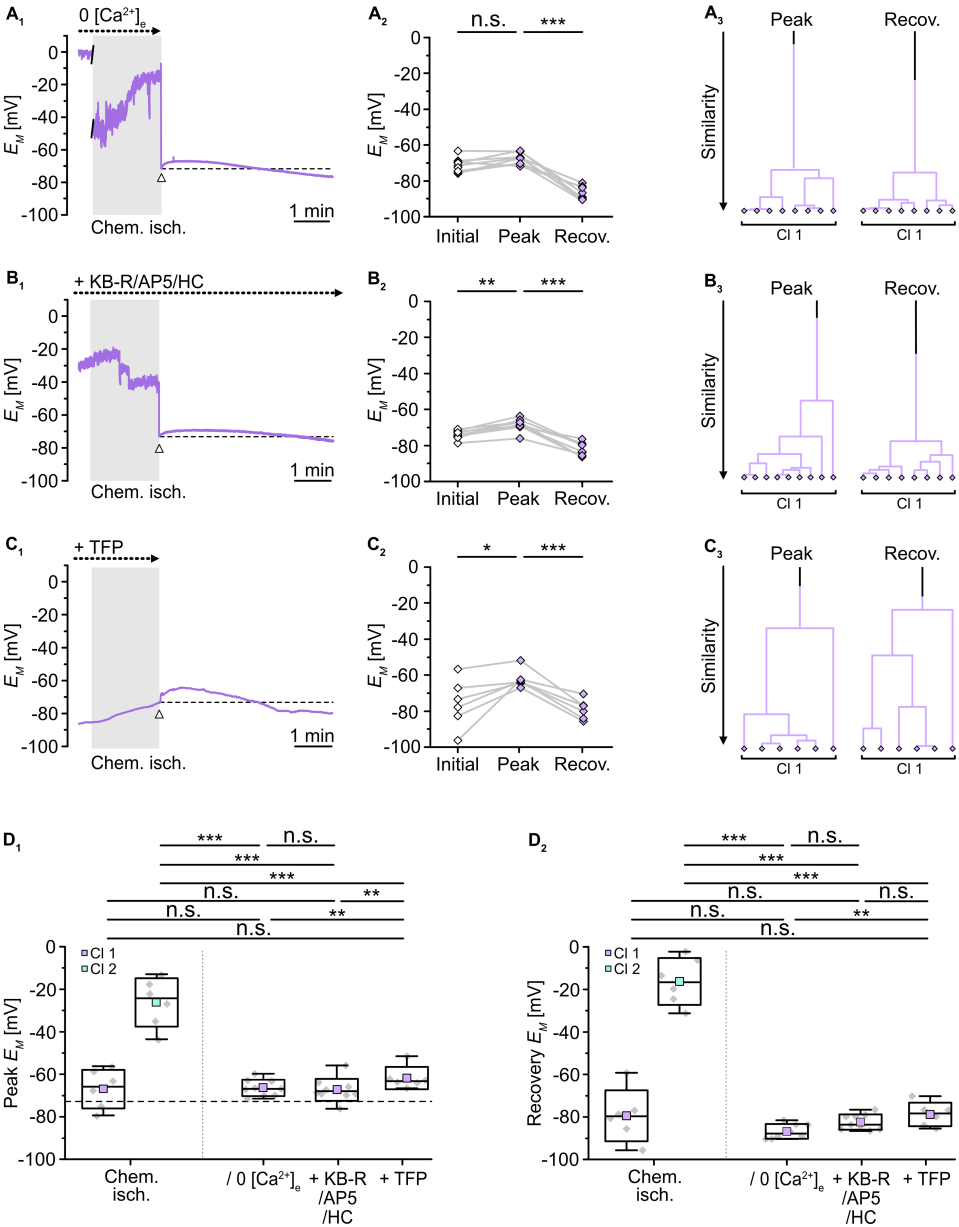
Role of [Ca^2+^]_i_ in alteration of gap junctional coupling. **(A–C)** Isopotentiality of astrocytes was recorded in 0 [Ca^2+^]_e_ **(A)**, with blockers of Ca^2+^ influx **(B)**, and with a blocker of calmodulin **(C)**. Gray bars show the periods when slices were exposed for 2 min to chemical ischemia. The dashed lines in **(A**_**1**_**–C**_**1**_**)** indicate the initial *E_M_* recorded upon membrane rupture (arrowhead). The dot plots in **(A**_**2**_**–C**_**2**_**)** illustrate changes in *E_M_* of all cells recorded. Data points derived from individual cells are connected by lines. The colored symbols reflect the results obtained from cluster analysis determining the similarity of individual data points. Whereas cluster analysis revealed the presence of two clusters in control (see Figure 2B_4_), only one cluster was detected upon reduction of Ca^2+^ influx **(A**_**3**_**,B**_**3**_**)** and inhibition of calmodulin **(C**_**3**_**)**. **(D)** Summary of results. The box plots depict peak *E_M_* **(D**_**1**_**)** and recovery **(D**_**2**_**)** in isopotentiality recordings in response to chemical ischemia (see Figure 2B) and in cells exposed to reduction of Ca^2+^ influx and during inhibition of calmodulin. Note that all manipulations reduced the number of clusters to one, which exhibited a peak *E_M_* similar to that of cluster 1 control cells. The box plots show individual data points (gray diamonds), mean (squares), median (horizontal line), SD (box), and min/max (whiskers). Significance levels are indicated by asterisks: *p < 0.05, **p < 0.01, ***p < 0.001. n.s., not significant.

To test whether a reduction in the overall Ca^2+^ load and/or an inhibition of calmodulin would affect the observed changes in gap junctional coupling upon brief metabolic stress, we next performed isopotentiality measurements. In 0 [Ca^2+^]_e_, chemical ischemia for 2 min caused a peak depolarization of astrocyte *E_M_* to -66.6 ± 3.8 mV (*n* = 9/9/3) and data were grouped into a single cluster (Figure 7A_3_). Notably, this peak *E_M_* was indistinguishable from that of the former cluster 1 (*p* = 0.756) and much more negative than that of the former cluster 2 (*p* = 3 × 10^−7^; Figure 7D_1_). In the following, all cells showed a complete recovery and a subsequent hyperpolarization within the recording period of 5–30 min (Figures 7A_2_,D_2_), similar to the hyperpolarization observed in cell-attached recordings (*cf.* Figures 2A_1,2_).

As observed upon removal of extracellular Ca^2+^, combined inhibition of NCX, NMDA receptors, and TRPV4 channels during chemical ischemia led to a peak *E_M_* of -68.7 ± 3.4 mV (*n* = 9/9/3, *p* = 1 × 10^−4^) and data were affiliated to a single cluster (Figure 7B). Compared to chemical ischemia without these blockers, peak *E_M_* was again indistinguishable from that of the former cluster 1 (*p* = 0.651) and exhibited a much more negative peak *E_M_* than that of the former cluster 2 (*p* = 1 × 10^−7^; Figure 7D_1_). Thereafter, all astrocytes completely recovered and in addition hyperpolarized (Figure 7D_2_).

Finally, in the presence of TFP, chemical ischemia led to a peak *E_M_* of -61.8 ± 5.3 mV (*n* = 6/6/5, *p* = 0.031) with the data being clustered into a single group (Figure 7C). This cluster again was indistinguishable from the former cluster 1 (*p* = 0.377) and exhibited a much more negative peak *E_M_* compared to the former cluster 2 (*p* = 0.004; Figure 7D_1_). Like in the two previous manipulations, all cells showed a complete recovery followed by hyperpolarization (Figure 7D_2_). Thus, removal of extracellular Ca^2+^, inhibition of Ca^2+^ entry, or the inhibition of calmodulin resulted in the loss of cluster 2 leaving only astrocytes with a less depolarized peak *E_M_*, which reflects a significant rescue from chemical ischemia-induced uncoupling.

In summary, our results again point towards the existence of two groups of astrocytes, this time differing in the magnitude of their [Ca^2+^]_i_ increase upon transient chemical ischemia. Moreover, they suggest that cells which experience a large peak increase in [Ca^2+^]_i_ also show rapid and sustained uncoupling. Our results further imply that astrocytes showing only a moderate change in [Ca^2+^]_i_ do not uncouple and recover quickly. This indicates that the observed reduction in gap junctional coupling in response to brief metabolic stress depends on the magnitude of the concomitant [Ca^2+^]_i_ increase in astrocytes. Finally, our data indicate that the gap junction modulator calmodulin mediates the observed Ca^2+^-dependent astrocyte uncoupling.

## 4. Discussion

In the present study, we employed syncytial isopotentiality recordings and quantitative ion imaging in acute mouse neocortical tissue slices to analyze the effects of brief metabolic stress on astrocyte gap junctional coupling. Our results show that 50% of neocortical astrocytes exhibited an immediate reduction in coupling. While we confirmed a general dependence of astrocytic gap junction coupling on pH_i_, the data suggest that the reduced gap junctional coupling upon brief metabolic stress was not mediated by concomitant (moderate) intracellular acidification. Attenuation of changes in [Ca^2+^]_i_, however, largely prevented uncoupling, indicating that it was dependent on the induced astrocytic Ca^2+^ load.

### 4.1. Probing astrocytic gap junctional coupling

As demonstrated here, loading a gap junction-permeable tracer into a single astrocyte via a patch-clamp pipette enables the subsequent visualization of neighboring coupled cells. Tracer coupling is a highly robust and informative approach to demonstrate the topography of networks (Wallraff et al., 2006; Houades et al., 2008; Augustin et al., 2016; Eitelmann et al., 2019). However, it typically requires a tracer loading time of at least 10–20 min and provides a more or less static view on astrocyte coupling. Besides neurobiotin which was used in the present study, there are fluorescent dyes that *per se* allow online monitoring of gap junctional transfer. However, these dyes less efficiently pass through gap junctions or even do not pass certain connexons, e.g., lucifer yellow comprises low permeability at gap junctions consisting of connexin 30 (Rackauskas et al., 2007; Abbaci et al., 2008; Stephan et al., 2021). Tracer coupling is thus less suited to address fast changes in coupling strength. In contrast, high temporal resolution can be obtained by electrophysiological techniques, and classical studies have provided fundamental insights into the properties of gap junctional coupling between astrocytes and/or other glial cells (Kettenmann and Ransom, 1988; Dermietzel et al., 1991; Giaume et al., 1991).

In the present study, we employed a patch-clamp-based approach in which gap junctional coupling is assessed via recording of astrocyte isopotentiality (Ma et al., 2016; Kiyoshi et al., 2018; Stephan et al., 2021). Here, the rapid exchange of K^+^ with neighboring cells is taken as a measure of the coupling strength of a given astrocyte. Previous reports established that the degree of depolarization of a given astrocyte induced by the K^+^-free pipette solution is inversely correlated to tracer coupling with neighboring cells (Zhong et al., 2023), which was roughly confirmed here. Our results confirm the applicability of this approach in cortical layer II/III astrocytes. In contrast to pyramidal neurons and putative NG2 cells, these astrocytes conferred isopotentiality, which could be attenuated by blocking gap junctions with CBX.

### 4.2. Brief metabolic stress induces rapid changes in astrocyte coupling strength

Several studies have demonstrated that prolonged energy deprivation results in uncoupling of astrocytes. For example, initial experiments on cultured rodent astrocytes revealed a strong reduction of coupling upon inhibition of oxidative phosphorylation for 16 h (Vera et al., 1996). Similarly, a strong decrease in astrocytic coupling strength was observed in hippocampal slices in response to at least 30 min of metabolic stress (Xu et al., 2010; Lee et al., 2016). Such manipulations mimic the sustained breakdown of energy metabolism in the core region of an ischemic stroke, which leads to a breakdown of ion gradients and massive cell damage and death (Rossi et al., 2007; Moskowitz et al., 2010; van Putten et al., 2021).

In the ischemic penumbra surrounding the core region, cells undergo repetitive waves of spreading depolarizations. These spreading depolarizations are characterized by transient accumulation of extracellular glutamate, cellular depolarization and acidification, loss of cellular ATP, and a reversible increase in [Na^+^]_i_ as well as [Ca^2+^]_i_ (Chuquet et al., 2007; Murphy et al., 2008; Pietrobon and Moskowitz, 2014; Dreier and Reiffurth, 2015; Rakers and Petzold, 2017; Rakers et al., 2017; Gerkau et al., 2018; Eitelmann et al., 2022). We have recently shown that exposing acute tissue slices to brief chemical ischemia, induced by a 2-minute perfusion with inhibitors of glycolysis and oxidative phosphorylation, mimics the transient changes in [Na^+^]_i_ and [Ca^2+^]_i_ seen during passage of a spreading depolarization in the mouse brain after middle cerebral artery occlusion (Gerkau et al., 2018; Meyer et al., 2022). In addition, this metabolic stress leads to a transient [K^+^]_e_ increase, a transient depolarization of astrocytes, a transient decrease in their ATP content, and transient extra- and intracellular acidification (Gerkau et al., 2018; Eitelmann et al., 2022; Pape and Rose, 2023), all further characteristics of spreading depolarizations as described above.

Here, we employed this *in situ* model to address possible changes in gap junctional coupling in response to chemical ischemia. Notably, chemical ischemia leads to various other changes in astrocytes and the extracellular space. In our former work, we have, e.g., shown that extracellular K^+^ transiently increases by about 2 mM (Eitelmann et al., 2022). The [K^+^]_e_ increase was accompanied by a transient change in astrocyte *E_M_*, which in the present study amounted to a depolarization by about 14 mV, indicating a redistribution of K^+^. A similar average depolarization to about -70 mV (across both clusters) was also seen in isopotentiality recordings at the end of the 2-minute period of chemical ischemia when cells were still held in the cell-attached mode, again most likely reporting the change in *E_M_* induced by redistribution of K^+^. Notably, when opening the patch to expose cells to the 0 K^+^ intracellular saline, we observed no significant further depolarization in 50% of the astrocytes, whereas the other 50% exhibited an additional depolarization adding to that recorded in cell-attached mode amounting to a peak *E_M_* of about -25 mV. This is significantly more positive than (a) the membrane depolarization in control isopotentiality recordings (about -80 mV) and (b) the depolarization induced by transient chemical ischemia (about -70 mV). We conclude from these observations that the stronger depolarization observed in 50% of cells upon dialysis with 0 K^+^ reflects a reduced isopotentiality due to a reduced exchange of K^+^ via the gap junctional syncytium.

The pronounced chemical ischemia-induced decrease in coupling strength seen in half of the astrocytes evolved already within 10–20 s after a 2-minute perfusion with metabolic inhibitors. This substantial decrease in coupling strength is similar to that found upon 5 min of oxygen–glucose deprivation in HeLa cells expressing Cx43, a major component of astrocytic gap junctions (Sahu et al., 2014). Upon reperfusion with standard saline and wash-out of metabolic inhibitors, respectively, half of the cells quickly repolarized indicating recoupling. The other half of astrocytes, in contrast, exhibited a depolarization that was maintained as long as the recordings lasted (i.e., for at least 5 min to up to 20 min), indicating more persistent uncoupling. This divergence in their ability to recover indicates a different susceptibility of astrocytes to metabolic stress. Such differential susceptibility was also reported in neocortical slices exposed to oxygen–glucose deprivation, another *in situ* model of the ischemic brain. In their study, Benesova et al. (2009) identified two astrocyte groups of approximately equal size that differed in their response to energy deprivation based on measurements of *E_M_* and cell swelling. Among others, they found differences in astrocytic K_ir_4.1 expression as well as taurine levels between the two groups and speculated that these were causal for the observed heterogeneity (Benesova et al., 2009). Recent single-cell RNA sequencing data furthermore indicated the presence of five astrocyte subpopulations in the mouse forebrain, two of which (AST2 and 3) were dominant in layers II/III (Batiuk et al., 2020). These two subtypes mainly differed in transcripts related to glutamatergic versus GABAergic neurotransmission. This eventually points towards a differential responsiveness to glutamate, which is reminiscent of an increased susceptibility to glutamate-mediated injury as suggested here (Batiuk et al., 2020).

### 4.3. Causes and possible consequences of astrocyte uncoupling

Gap junctional coupling depends on various factors, e.g., expression, trafficking, and de−/phosphorylation of connexins as well as changes in pH_i_ and [Ca^2+^]_i_ (Giaume et al., 2021). Here, we found that acidification of astrocytes by 0.45 pH units using low [HCO_3_^−^]_e_ saline indeed caused an immediate decrease in isopotentiality in all astrocytes investigated, indicating a rapid modulation of gap junctional coupling by such changes in pH_i_. Astrocyte uncoupling was visualized in a tracer coupling study after prolonged administration of low [HCO_3_^−^]_e_ saline (Wallraff et al., 2006). However, acidification can generally interfere with protein function, by reducing, e.g., conductivity of K_ir_ channels (Yang and Jiang, 1999; Xu et al., 2000). Under resting conditions, these channels mediate K^+^ efflux contributing to the highly negative *E_M_* of astrocytes. In turn, inhibition of K_ir_ channels causes a strong depolarization of astrocytes (Seifert et al., 2009; Stephan et al., 2012). Accordingly, we cannot finally exclude that the acidification-induced reduction in astrocyte isopotentiality upon the 5-min administration of low [HCO_3_^−^]_e_ saline could be partially due to pH-mediated inhibition of K_ir_ channels.

In contrast, acidifying astrocytes by only 0.26 pH units, which mimicked the acidification associated with a 2-minute chemical ischemia, had no effect on astrocyte isopotentiality and accordingly on astrocyte coupling. However, this does not *per se* indicate that there cannot be a synergistic effect of the pH_i_ change during chemical ischemia, in which acidification and Ca^2+^ load occur simultaneously. A synergistic inhibition of gap junctions by changes in pH_i_ and [Ca^2+^]_i_ was described for cardiac myocytes (Burt, 1987; White et al., 1990). In Novikoff hepatoma cells and astrocytes, however, even an intracellular acidification to less than pH 6.1 and 6.4, respectively, - i.e. a much stronger acidification compared to what we observed during a 2-minute chemical ischemia (pH 7.07; this study; Eitelmann et al., 2022) -, was not sufficient to increase Ca^2+^-dependent uncoupling (Lazrak and Peracchia, 1993; Cotrina et al., 1998). Together, this strongly indicates that the uncoupling of astrocytes upon brief metabolic stress observed in the present study was primarily independent from the (moderate) intracellular acidification induced by chemical ischemia.

Metabolic inhibition causes an immediate Ca^2+^ load (this study; Gerkau et al., 2019) and gap junctions close in response to elevation of [Ca^2+^]_i_ (Spray et al., 1982; Muller et al., 1996; Cotrina et al., 1998; Lurtz and Louis, 2007; Giaume et al., 2021). In the present study, Ca^2+^ imaging experiments revealed two clusters of astrocytes: cells of cluster 1 showed a peak [Ca^2+^]_i_ increase of less than 200 nM, whereas the [Ca^2+^]_i_ increase in cells of cluster 2 was higher than ~250 nM. Indeed, a [Ca^2+^]_i_ of ~250 nM was shown to inhibit gap junctional communication in Cx43 expressing N2a cells and cardiomyocytes (Noma and Tsuboi, 1987; Xu et al., 2012). Furthermore, elevated [Ca^2+^]_i_ in the lower nM range was shown to also inhibit astrocyte gap junctions (Enkvist and McCarthy, 1994). Notably, dampening the Ca^2+^ influx from the extracellular space not only reduced peak [Ca^2+^]_i_ changes to less than 200 nM, but also reduced the number of clusters from two to one. Most importantly, this dampening of Ca^2+^ load correlated with reduced astrocyte uncoupling. In addition, the chemical ischemia-induced uncoupling was prevented upon inhibition of calmodulin. It is well established that this Ca^2+^-binding protein modulates gap junction communication (Peracchia et al., 1983; Peracchia, 1984, 1987) and in more recent years, different calmodulin binding domains were found at the cytoplasmic loop of Cx43 (Zhou et al., 2007; Myllykoski et al., 2009; Xu et al., 2012). We conclude from our experiments that the threshold for the Ca^2+^/calmodulin-dependent closure of astrocytic gap junctions upon brief metabolic stress was about 200–300 nM. This value, however, must be considered with caution, because [Ca^2+^]_i_ changes were determined in somata and may differ in fine astrocyte processes, where most connexins are expressed.

Inhibition of calmodulin with TFP also reduced the Ca^2+^ load. This is similar to observations in the ischemic liver and in glioma cells, where TFP applied in the μM range reduced Ca^2+^ levels (Chien et al., 1977; Wen et al., 2018). Further, chlorpromazine, another phenothiazine like TFP also inhibiting calmodulin, reduced Ca^2+^ levels in the ischemic heart as well in hippocampal neurons after spreading depression (Chien et al., 1979; Balestrino and Somjen, 1986). Here, the Ca^2+^ load might be counteracted by several different mechanisms, like, e.g., TFP-mediated maintenance of Ca^2+^-ATPase activity (Souza dos Santos et al., 2007) and/or increasing the Ca^2+^ binding (and buffer) capacity of calmodulin (Tanokura and Yamada, 1985; Feldkamp et al., 2010). Whether any of the two mechanisms is responsible for the observed reduction in the chemical ischemia-induced Ca^2+^ elevation, needs further investigation.

We have strong experimental evidence that the reduced gap junctional coupling seen in 50% of the astrocytes during chemical ischemia resulted from a Ca^2+^-dependent activation of calmodulin. However, there might be additional mechanisms contributing to astrocyte uncoupling. For example, gap junctional coupling in astrocytes was shown to be regulated by neuronal activity (Rouach et al., 2000, 2008). In our experiments, all manipulations also effect neighboring neurons. Upon metabolic inhibition, neurons in the somatosensory cortex undergo a substantial loss of ATP and concomitant Na^+^ and Ca^2+^ load via NMDA receptors and TRPV4 channels (Gerkau et al., 2018; Pape and Rose, 2023). In the following, neurons might interact with astrocytes and indirectly induce uncoupling. Nonetheless, our data clearly show that there is a direct and strong correlation between astrocytic Ca^2+^ load and the reduced coupling of astrocytes and that a reduction in the Ca^2+^ influx or pharmacological inhibition of the Ca^2+^-dependent gap junction modulator calmodulin prevents uncoupling.

The functional consequences of a Ca^2+^-dependent reduction in astrocyte coupling upon brief metabolic stress are currently unclear. This is mainly due to conflicting results regarding the functional role of astrocyte gap junctional coupling in the ischemic brain, which may be at least partly related to opposing actions mediated by gap junction channels versus hemichannels (Contreras et al., 2004; Rovegno and Saez, 2018; Liang et al., 2020; Giaume et al., 2021). On the one hand, there are reports showing that Cx43 knock-out mice exhibit larger infarct volumes, suggesting a protective role of gap junctions (Siushansian et al., 2001; Nakase et al., 2003). On the other hand, some studies describe a gap junction-mediated expansion of cell injury (Lin et al., 1998; Lauritzen et al., 2011). In addition, further studies demonstrated that reduced expression or inhibition of Cx43 results in decreased neuronal death and infarct volume (Rawanduzy et al., 1997; Rami et al., 2001; Li et al., 2015). Similarly, the role of astrocytic gap junctional coupling in the ischemic penumbra is debated. There is evidence that they promote the propagation of spreading depolarizations (Nedergaard et al., 1995; Seidel et al., 2016; EbrahimAmini et al., 2021). However, there are also findings that contradict this hypothesis (Theis et al., 2003; Enger et al., 2015).

Gap junctional coupling carries and supports the propagation of intercellular astrocytic Ca^2+^ waves (Haydon, 2001; Verkhratsky and Nedergaard, 2018). The dissemination of an [Ca^2+^]_i_ elevation and of other signaling molecules from an injured astrocyte to its neighbors via gap junctions has been proposed to spread the damage in the syncytium (Contreras et al., 2004; Decrock et al., 2009; De Bock et al., 2014). In the present study, we found that brief metabolic stress led to rapid, substantial uncoupling of astrocytes experiencing a large [Ca^2+^]_i_ increase. During passage of spreading depolarizations in the ischemic penumbra, such Ca^2+^-dependent uncoupling would separate cells from each other thereby preventing additional Ca^2+^ loads arising from neighboring cells. Notably, a recent study showed that dampening of spreading depolarization-evoked astrocytic Ca^2+^ signals results in decreased extracellular glutamate accumulation, reduced spreading depolarization frequency and burden as well as increased neuronal survival after stroke in a mouse model of focal ischemia (Rakers and Petzold, 2017). Based on our study performed in acute tissue slices, we thus propose that disconnecting parts of the astrocyte network may exert a protective role, helping to confine and reduce spreading of cellular damage in the ischemic penumbra *in vivo*.

## Data availability statement

The raw data supporting the conclusions of this article will be made available by the authors, without undue reservation.

## Ethics statement

The animal study was approved by Dr. Martin Sager and Dr. Eva Engelhardt from the Animal Welfare Office of the Animal Care and Use Facility of the Heinrich Heine University Düsseldorf. The study was conducted in accordance with the local legislation and institutional requirements.

## Author contributions

JS, CRR, and SE designed the experiments and wrote the manuscript. SE, KE, LP, and JS performed the experiments and analyzed the data. All authors approved the final version.
